# More Fixations in Static Facial Regions During Emotion Recognition Among TLE Patients With Severe Depression

**DOI:** 10.1111/cns.70636

**Published:** 2025-10-29

**Authors:** Haojun Yang, Qiong Zhang, Kailing Huang, Li Feng

**Affiliations:** ^1^ Department of Anesthesiology Xiangya Hospital of Central South University Changsha China; ^2^ National Clinical Research Center for Geriatric Disorders, Xiangya Hospital, Central South University Changsha China; ^3^ Department of Neurology Xiangya Hospital of Central South University Changsha China

**Keywords:** depression, emotion recognition, eye‐tracking, temporal lobe epilepsy

## Abstract

**Aim:**

To explore the emotion recognition (ER) abilities among temporal lobe epilepsy (TLE) patients with different levels of depression.

**Methods:**

This observational study was conducted during December 2021 and November 2022, including 71 healthy controls (HCs) and 93 TLE patients. Based on the scores of the Beck Depression Index, the patients were divided into four groups (no/mild/moderate/severe depression). All participants finished the Faux pas task (FPT), watched the dynamic facial expression task and recognized the emotion (anger, disgust, happiness, or sadness) with the recording of eye movements. The accuracy of ER was recorded. The percentage of fixation in interested areas (IA) and fixation counts in IA were selected and analyzed.

**Results:**

TLE patients without depression had significantly lower FPT scores (*p* = 0.001) and accuracy ratios of dynamic facial ER (*p* = 0.039) than HCs. Patients with severe depression had significantly more fixation percentages and fixation counts in the nose than patients with no/mild/moderate depression (*p* < 0.05).

**Conclusion:**

The recording of eye movement provides more accurate and objective biobehavioral indicators for distinguishing TLE patients with severe depression, which has great practical significance for early monitoring and intervention.

## Graphical abstract

Eye‐tracking data revealed distinct attentional patterns: TLE patients with severe depression exhibited abnormal fixation preferences, disproportionately focusing on static facial features (e.g., nose) rather than dynamic, emotionally informative regions (e.g., eyes and mouth).

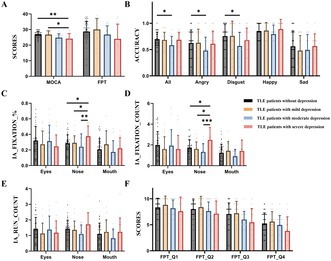

## 1 Introduction

Temporal lobe epilepsy (TLE), one of the most common chronic neurological diseases, is often complicated by social cognition impairments [1]. Social cognition refers to the psychological processes that are involved in the perception, encoding, storage, retrieval and regulation of information about other people and ourselves. Social cue perception, experience sharing, inferring other people's thoughts and emotions and managing emotional reactions to others are all included in the above processes [2, 3]. The abilities to identify facial expressions from other people and to attribute mental states to others refer to the preserved function of the temporal lobes [4]. Previous studies have demonstrated social cognitive deficits, especially emotion recognition (ER) impairments, in TLE from the evidence of neuropsychological scales and behavior tests [4, 5].

Although ER deficits in TLE patients have been widely reported, critical methodological limitations persist in current research. First, conventional assessments relying on neuropsychological scales or behavioral tasks are inherently susceptible to subjective biases from participants, limiting their reliability. More critically, these approaches fail to elucidate the underlying neural mechanisms driving ER impairments. Eye‐tracking technology has emerged as a powerful tool to address these gaps, offering an objective, quantifiable measure of cognitive dysfunction through precise analysis of gaze patterns. Our recent eye‐tracking study found TLE patients exhibited pronounced ER deficits, particularly for negative emotions, which may be attributable to their reduced fixation on faces, and especially the eyes [6]. These findings indicate that eye‐tracking technology can provide real‐time process‐based biomarkers of ER and is a complementary paradigm to traditional behavioral assessments.

Depressive symptoms are one of the most prevalent psychiatric comorbidities in TLE. Affected by traditional and superstitious views that TLE is caused by evil spirits and can be transmitted from the patient, patients with TLE often suffer from severe stigma and discrimination in China, especially in rural areas [7]. Many depressive symptoms are derived from concerns about discrimination and unfair treatment by those around them. Consequently, it is often difficult for clinicians to identify TLE patients with depressive symptoms, as affected individuals may conceal their emotional distress to avoid further stigmatization. Moreover, the current clinical landscape lacks reliable objective biomarkers for the early identification of depressive symptoms in this vulnerable population.

Previous studies have found that ER is significantly related to trait anxiety‐depression and neuroticism [8, 9]. So are there significant differences in ER abilities among TLE patients with or without depression, as well as with different levels of depression? No relevant studies have been seen yet. Based on the above evidence, this study aimed to evaluate the ER abilities among TLE patients with different levels of depression by using scales, a video‐based ER task and Eye‐Tracking technology. We hope to find ER‐related changing characteristics in TLE patients with depressive symptoms to facilitate early identification and intervention in clinical practice.

## 2 Methods

This study was approved by the ethics committees of Xiangya Hospital, Central South University. We clearly explained that the aim of this study and written informed consents were obtained from participants before proceeding with the progress.

### 2.1 Participants

Patients with TLE who visited the department of Neurology, Xiangya Hospital, Central South University between December 2021 and November 2022 were enrolled in this study. TLE was diagnosed according to the International League Against Epilepsy criteria [10]. Age‐ and sex‐matched healthy volunteers without depression served as healthy controls. Participants with chronic physical or psychiatric disease, alcohol addiction, caffeine or psychotropic drugs, and those during pregnancy or lactation were excluded. In addition, participants were required to be at least primary school graduates to ensure full understanding and effective filling of the evaluation.

### 2.2 Neurobehavioral and Psychological Assessment

#### 2.2.1 Evaluation of General Cognition

We applied Montreal Cognitive Assessment—the Beijing version (BJ‐MoCA) to evaluate the general cognitive function of participants, which has demonstrated an excellent sensitivity of 90.4%, and a fair specificity (31.3%) [11].

#### 2.2.2 Evaluation of Mental Status

The Back Depression Inventory (BDI) was used to evaluate the mental status of the participants, which has been widely used in the Chinese population [12], especially patients with epilepsy [13]. The BDI consists of 21 items reflecting subjective and vegetative symptoms of depression, each item is rated on a 4‐point scale ranging from 0 (not at all) to 3 (severely). Total scores range from 0 to 63 and are categorized into four levels of severity: no depression (total score below 13), mild depression (total score, 14–19), moderate depression (total score, 20–28) and severe depression (total score ≥ 29) [14].

#### 2.2.3 Theory of Mind

We applied the Faux pas task (FPT) to evaluate the Theory of Mind; the subjects were required to read a short story and answer four questions to recognize the true intention of the person in the story. The first question was to recognize if someone had said something he/she should not have said, the second question was concerning who made the remark, the third question was about the reason why he/she should not make the remark, the fourth question was to speculate whether he/she said it on purpose or not. There were 10 stories in total; one point was given for each correct answer, the full score of FPT was 40. If the subjects answered no to the first question, then they tended to the control question to determine whether they understood the story and continued the test. The 3‐month test–retest reliability among Chinese was 0.83, and the interrater reliability was 0.76 [15].

### 2.3 Eye‐Tracking Materials and Procedures

#### 2.3.1 Stimuli

The stimuli included dynamic facial expression video provided by the 3D Dynamic Facial Expression Database of Binghamton University (https://www.cs.binghamton.edu/~lijun/Research/3DFE/3DFE_Analysis.html). The dynamic facial expression task comprised six individuals (3 males, 3 females) from an Asian country, including four basic emotions: anger, disgust, happy and sad; each facial expression was presented six times. The duration of the video clip was 2–3 s.

#### 2.3.2 Procedure

Participants were seated in a quiet room at a 45 cm viewing distance from the computer screen, and at the height where their eyes fell on the middle of the computer screen. The participants' eye gaze was continuously recorded while performing the task. The order of emotion occurrence in the dynamic facial expression task was random. The participants observed the videos and had to select the corresponding emotion label for each task from four choices displayed on the screen. Once the choice was made, the procedure would proceed to the next trial. Ahead of the formal experiment, there would be two exercises to ensure the participants understood the experiment. The practice videos would no longer appear in the subsequent procedure.

#### 2.3.3 Eye‐Tracking Data Collection

Eye‐Tracking data were collected using the EyeLink 1000 Plus eye tracker—monocular remote mode—running at 500 Hz and DataViewer software. As we used remote mode in which the participants' chins were not restrained, we asked the participants not to move their upper bodies as much as possible. The standard 13‐point calibration ensures the precision and accuracy of the data. The participants who failed the calibration would not be included in the subsequent experiments. Areas of interest (AOIs) were drawn by Data Viewer; the AOIs of the dynamic facial expression task included the eyes, nose, and mouth.

#### 2.3.4 Eye‐Tracking Indicators

Eye‐Tracking data were collected at a sampling rate of 500 Hz monocular and analyzed with DataViewer (version 4.2). After preprocessing the Area of Interests (AOIs) report, we extracted Eye‐Tracking indicators: fixation count, fixation% and run count. Fixation count refers to the total number of fixations falling in the interest area. Fixation% refers to the percentage of all fixations in a trial falling in the current interest area. Run count refers to the number of times the Interest Area was entered and left (runs).

### 2.4 Statistical Analysis

Statistical analysis was performed using the SPSS software package (ver. 26.0; SPSS Inc., Chicago, Illinois, USA). Characteristics of participants were compared using chi‐squared analyses for categorical variables. All data were subject to tests for normality by the Shapiro–Wilk test. Independent Sample *t*‐test was used for the continuous variables that passed the normality test, and the data were presented as means and standard variations (Mean ± SD), while the nonparametric equivalent (Mann–Whitney *U* test) was used for continuous variables that failed the normality test and the data were presented as median and interquartile range. In subgroup analysis, we used one‐way ANOVA to compare the means of four sample groups to see if there was a significant difference and used Bonferroni correction to reduce the risks of false positives. Spearman's correlation analysis was used to determine correlations between the memory scale assessment, the eye‐ tracking indicators, and imaging features. A two‐tailed *p*‐value was calculated for all tests and *p*‐values of less than 0.05 were considered statistically significant.

## 3 Results

### 3.1 Participant Characteristics

A total of 71 healthy controls without depression and 93 patients with TLE were involved in this study. According to the BDI scores, patients with TLE were divided into four groups: TLE patients without depression (score below 13), TLE patients with mild depression (score 14–19), moderate depression (score 20–28) and severe depression (score ≥ 29). No significant differences were found between each group (Table 1).

**TABLE 1:** Demographic characteristics of the study population.

		Group 0	Group 1	Group 2	Group 3	Group 4	P_01_/P_12_/P_13_/P_14_/P_23_/P_24_/P_34_
*N*		71	43	18	17	15	—
Age (years)		26.42 ± 6.388	27.88 ± 10.219	33.28 ± 13.122	29.76 ± 10.814	30.40 ± 11.891	0.349/0.090/0.530/0.435/0.395/0.518/0.875
Gender (female/male)		38/33	25/18	9/9	12/5	7/8	0.631/0.559/0.371/0.442/0.214/0.849/0.169
Education level	Junior high school	18	11	5	4	2	0.978/0.859/0.869/0.327/0.774/0.312/0.461
	Senior high school	22	13	5	7	7	
	Undergraduate	25	17	6	6	6	
	Postgraduate	6	2	2	0	0	
Marital status (single/married)		38/33	23/20	9/9	9/8	8/7	0.997/0.804/0.969/0.992/0.862/0.849/0.982
Duration (years)		—	8.42 ± 6.797	9.79 ± 11.006	12.08 ± 11.602	14.60 ± 12.182	NA/0.282/0.556/0.340/0.150/0.096/0.712
Onset age (years)		—	19.48 ± 11.196	22.88 ± 11.034	17.64 ± 9.924	16.26 ± 11.061	NA/0.555/0.135/0.079/0.554/0.243/0.553
Seizure frequency (per month)		—	4.20 ± 4.818	2.45 ± 2.694	2.10 ± 1.236	4.16 ± 4.302	NA/0.076/0.083/0.710/0.628/0.244/0.099
Number of ASMs		—	1.51 ± 1.202	1.61 ± 1.092	2.00 ± 1.369	2.20 ± 1.568	NA/0.763/0.178/0.084/0.358/0.214/0.703
Laterality (left/right)		—	22/21	10/8	11/6	9/6	NA/0.754/0.342/0.555/0.581/0.797/0.784

*Note:* Group 0 represents healthy controls without depression, and groups 1–4 represent TLE patients with no/mild/moderate/severe depression in turn, and *p*_01_ means *p*‐values between group 0 and 2. The other *p*‐values are similar to the above description.

### 3.2 Comparison of Healthy Controls and TLE Patients Without Depression

#### 3.2.1 Cognitive Levels and Mental State

**FIGURE 1:**
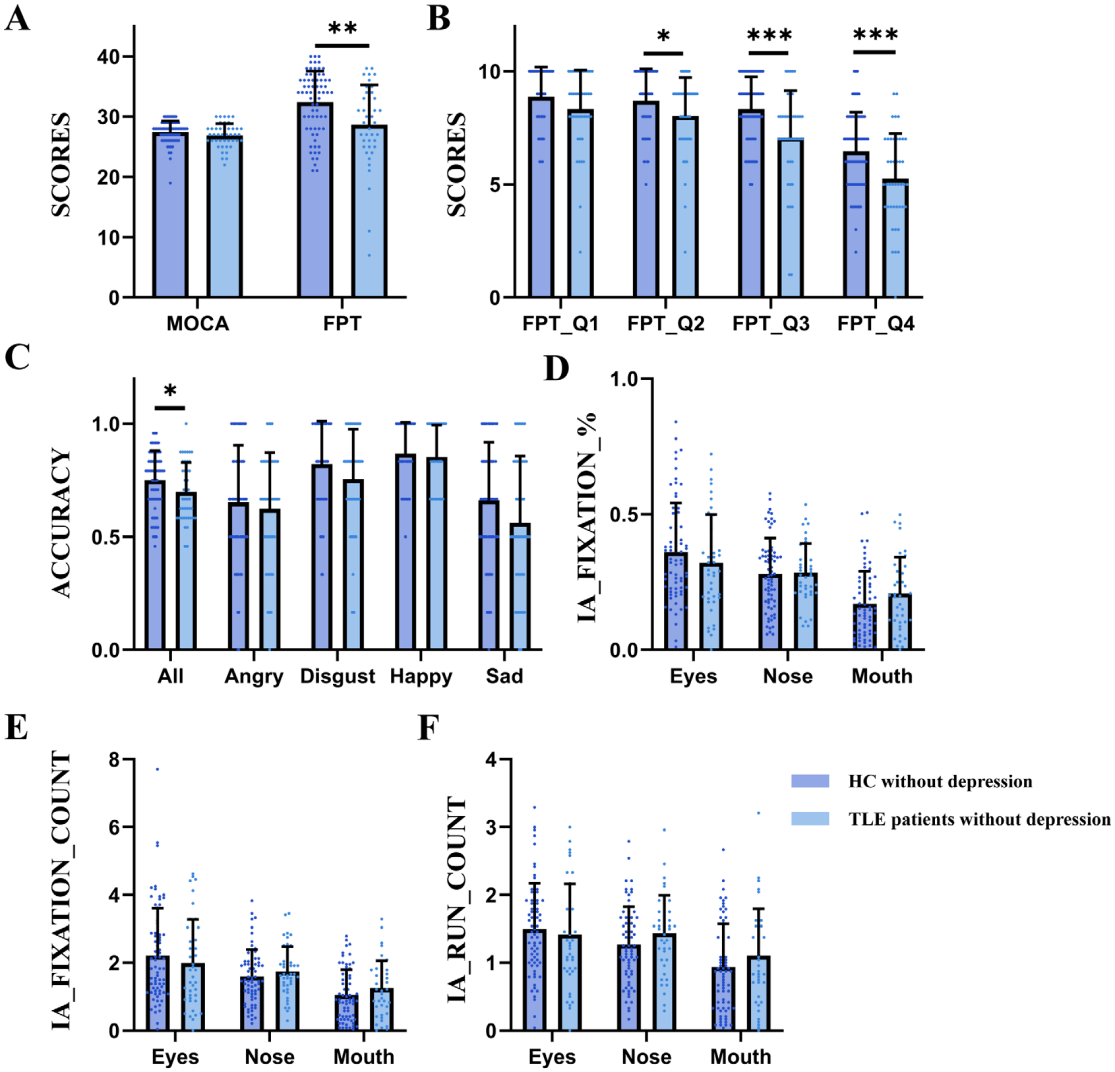
The results of scales, emotion recognition tasks and eye movements among healthy controls and TLE patients without depression. * represents *p*‐value < 0.05, ** represents *p*‐value < 0.01, *** represents *p*‐value < 0.001. Abbreviations: IA: interested areas; HC: healthy controls; TLE: temporal lobe epilepsy.

There were no significant differences in the scores of MoCA between healthy controls (26.86 ± 1.983) and TLE patients without depression (27.42 ± 1.887, *p* = 0.133) (Figure 1A).

#### 3.2.2 Faux Pas Task

The total score of FPT among TLE patients without depression (28.67 ± 6.582) was significantly lower than controls (32.36 ± 5.196, *p* = 0.001). As shown in Figure 1B, the scores of questions 2–4 among patients were also significantly lower than controls (*p* = 0.025, < 0.001, < 0.001, respectively).

#### 3.2.3 Dynamic Facial Emotion Recognition

The accuracy among healthy controls (0.75 ± 0.130) was significantly higher than TLE patients without depression (0.69 ± 0.130, *p* = 0.039) (Figure 1C). No significant differences were found between the two groups in the percentage of fixation, fixation count and run count in IA (Figure 1D–F).

### 3.3 Comparison of TLE Patients With Different Levels of Depression

#### 3.3.1 Cognitive Levels

**FIGURE 2:**
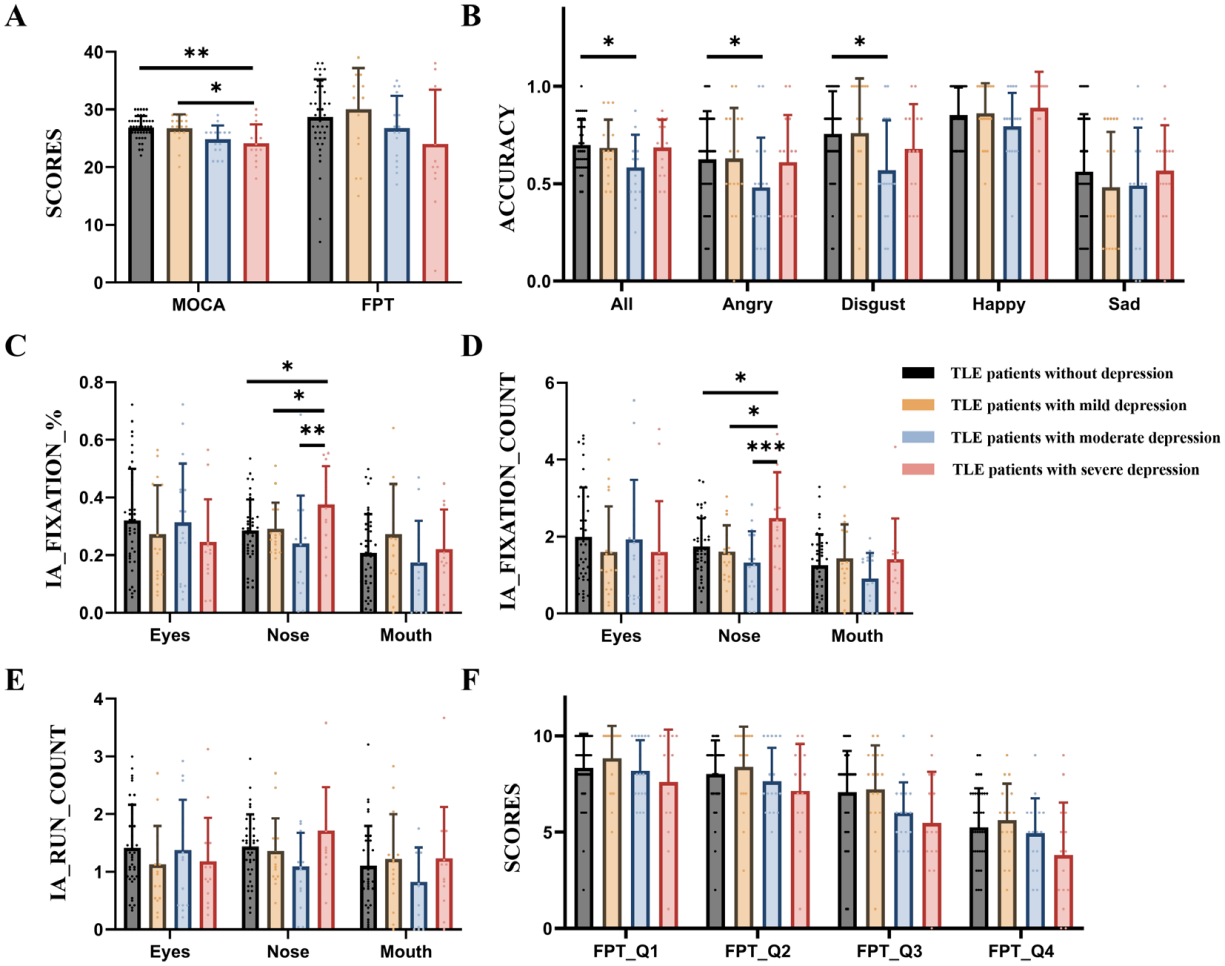
The results of scales, emotion recognition tasks and eye movements among TLE patients with different levels of depression. * represents *p*‐value < 0.05, ** represents *p*‐value < 0.01, *** represents *p*‐value < 0.001. Abbreviations: IA: interested areas; HC: healthy controls; TLE: temporal lobe epilepsy.

The score of MoCA among TLE patients with severe depression (24.13 ± 3.292) was significantly lower than that in patients with no depression (26.86 ± 1.983, *p* = 0.001) and mild depression (26.72 ± 2.421, *p* = 0.009) (Figure 2A).

#### 3.3.2 Faux Pas Task

As shown in Figure 2A,F, there were no significant differences among the four groups in terms of total scores and individual scores, indicating the FPT scale cannot distinguish whether TLE patients with depression have ER disorders.

#### 3.3.3 Dynamic Facial Emotion Recognition

1. Accuracy

The ER accuracy among patients without depression was 0.70 ± 0.130, which was significantly higher than that of patients with moderate depression (0.58 ± 0.169, *p* = 0.004), especially in distinguishing the emotion of “angry” (*p* = 0.048) and “disgust” (*p* = 0.034) (Figure 2B).

2 Eye‐Tracking indicators

As shown in Figure 2C, the percentage of fixation in the nose among patients with severe depression (0.38 ± 0.132) was significantly higher than that of patients with no (0.28 ± 0.107, *p* = 0.014), mild (0.29 ± 0.090, *p* = 0.049) and moderate (0.24 ± 0.148, *p* = 0.002) depression. In addition, the fixation count in the nose among patients with severe depression (2.48 ± 1.190) was all significantly more than that in patients with no (1.74 ± 0.734, *p* = 0.023), mild (1.61 ± 0.683, *p* = 0.020) and moderate (1.32 ± 0.812, *p* < 0.001) depression (Figure 2D).

3 Eye‐Tracking indicators in four emotions

**FIGURE 3:**
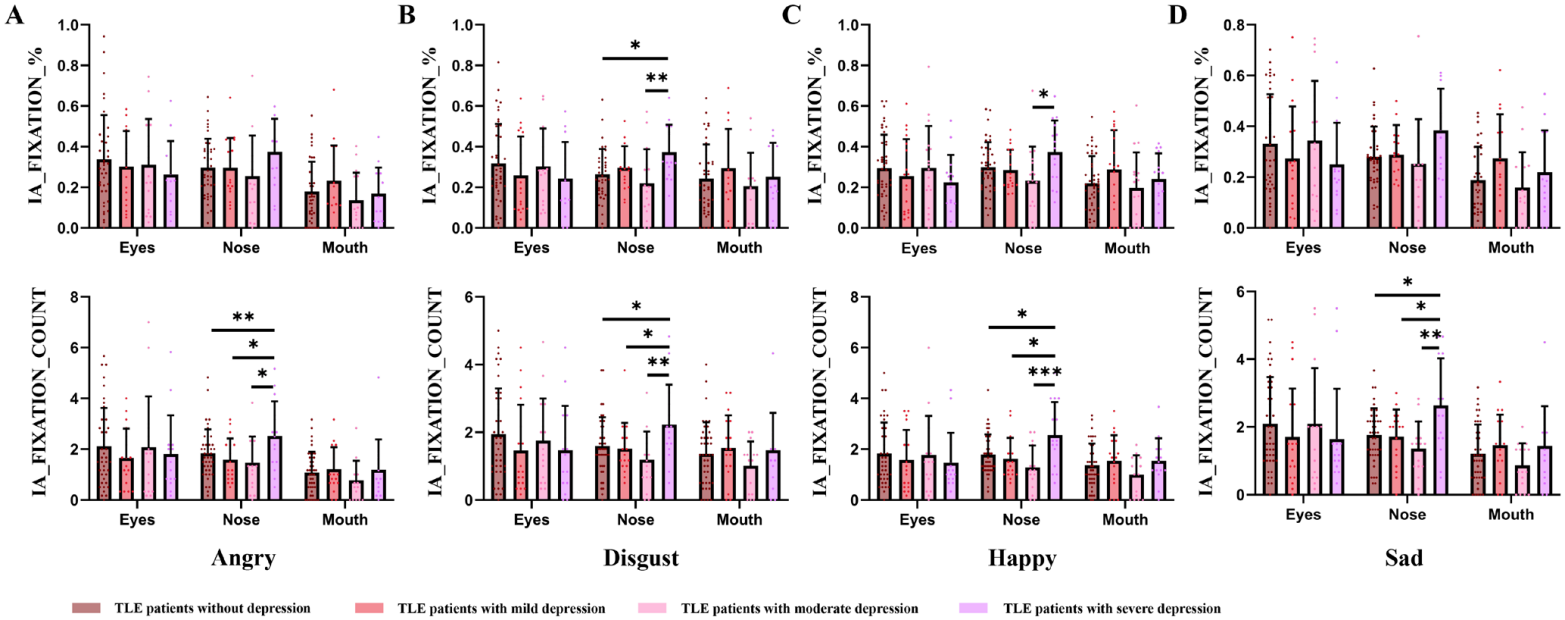
The eye‐tracking results of dynamic facial emotion recognition under four emotions (angry, disgust, happy, and sad) among TLE patients with different levels of depression. * represents *p*‐value < 0.05, ** represents *p*‐value < 0.01, *** represents *p*‐value < 0.001. Abbreviations: IA: interested areas; HC: healthy controls; TLE: temporal lobe epilepsy.

As shown in Figure 3, under the condition of four emotions, the fixation count in the nose among patients with severe depression was all significantly more than in the other groups. In addition, under the disgust emotion, the TLE patients with severe depression (0.37 ± 0.135) paid more fixation in the nose than patients with no (0.26 ± 0.125, *p* = 0.044) and moderate (0.22 ± 0.168, *p* = 0.009) depression. Specific values were shown in Table S1.

### 3.4 Correlation Analysis

Bivariate correlation analysis showed that the BDI value of TLE patients was negatively related to the cognitive level and positively related to the fixation percentage and count in the nose (Table S2). No significant correlations were found between the number of ASMs and other indicators (Table S3).

## 4 Discussion

This study represents the first investigation of ER abilities among TLE patients with different levels of depression. Our findings demonstrated that even nondepressed TLE patients showed significantly impaired performance compared to healthy controls, as evidenced by lower scores on FPT and reduced accuracy in dynamic facial recognition tasks. Interestingly, while FPT scores did not differ significantly among TLE patients with different levels of depression, eye‐tracking data revealed distinct attentional patterns: patients with severe depression exhibited abnormal fixation preferences, disproportionately focusing on static facial features (e.g., nose) rather than dynamic, emotionally informative regions (e.g., eyes and mouth).

The capacity to recognize emotions constitutes a fundamental social cognitive ability, with impairments potentially leading to significant psychological alterations and maladaptive social behaviors. Previous studies have demonstrated that TLE patients exhibit pronounced ER deficits, particularly for negative emotions including sadness, disgust, and fear [6, 16]. The early onset seizures with right or bilateral medial temporal dysfunction tend to cause severe deficits in recognizing facial expressions of emotions. The integrity of the temporal lobe and the interconnected brain regions [17] was thought to be the mechanism of ER. Patel et al. reported that schizophrenia patients with ER impairments display reduced visual attention to faces and emotionally salient facial features—areas that typically attract gaze in healthy individuals [18]. Our recent findings parallel these observations, demonstrating that TLE patients exhibit diminished focus on facial regions, particularly the eyes, during dynamic facial expression tasks. This shared attentional abnormality may reflect dysfunction within the amygdala network, representing a common pathophysiological mechanism across these disorders [19].

Eye‐tracking technology provides a real‐time, objective method for assessing ER abilities through precise measurement of ocular movements. Despite its potential, few studies have employed this technique to investigate ER impairments in patients with epilepsy. Huang et al. documented reduced fixation durations on eyes in focal epilepsy patients [6], while Gomez‐Ibanez et al. specifically observed this deficit in mesial TLE patients [20]. Although limited by small sample sizes, these consistent findings suggest impaired visual scanning of emotionally salient facial features in TLE. The amygdala is thought to be highly involved in the processing of ER and facial expressions. Neuroimaging studies demonstrate reduced amygdala activation during ER tasks in TLE patients [21, 22]. The amygdala contributes to the generation and propagation of epileptiform activity in TLE and also plays an important role in comorbid anxiety and depression experienced in the interictal phase [23]. Amygdala volumes were found to be highly related to comorbid depression among patients with bipolar disease or TLE [24]. Our study extends these findings by revealing distinct attentional patterns: TLE patients with severe depression exhibited significantly greater fixation on static facial features (e.g., nose) compared to those with no/mild/moderate depression. The above evidence indicated the epileptiform activity in TLE and comorbid depression were all related to the ER impairments, and the amygdala seemed to be the key nucleus involved in this process. In addition, we found the results of eye‐tracking instead of social scales, could distinguish TLE patients with severe depression from others. Therefore, the quantitative tracking of eye movement behavior in the process of ER can not only evaluate ER damage in TLE patients but also provide more accurate and objective biobehavioral indicators for assessing the levels of comorbid depression.

Finally, we strongly recommend implementing tailored psychosocial interventions (e.g., cognitive behavioral therapy [25]) delivered by trained healthcare professionals. These interventions should leverage clinicians' emotional intelligence competencies and social skills [26] to effectively alleviate emotional distress in both patients and their caregivers, and mitigate the substantial psychosocial burdens associated with epilepsy [27].

This study has several limitations that should be acknowledged. First, it was conducted in a single center, which may introduce geographical bias and limit the generalizability of the findings. Besides, TLE patients with moderate or severe depression who also meet the inclusion and exclusion criteria and agree to participate in this study, are relatively rare in clinical practice. So the respective sample size in TLE patients with moderate/severe depression was relatively small, which we hope to conduct in multicenter in the future. Next, this was a cross‐sectional descriptive study, thus lacking the ability to explore the improvement of ER impairment after the treatment of comorbid depression among TLE patients. Finally, the categories of ASMs may also affect the mental states of patients with TLE, which may have a potential impact on the statistical efficacy and reliability of the conclusions. Subsequent research should implement corresponding experimental designs to eliminate this potential interference.

## 5 Conclusion

This pioneering study represents the first systematic investigation of ER impairments in TLE patients with different levels of depression. Our findings demonstrate significant social cognitive deficits in TLE populations, with particularly notable impairments observed in severely depressed patients who exhibited abnormal visual attention patterns characterized by preferential fixation on static facial features during ER tasks. Eye‐tracking technology can provide the process indicators of cognition evaluation, which has great potential for clinical application in the future. The quantitative tracking of eye movement behavior in the process of ER can not only evaluate ER damage in TLE patients but also provide more accurate and objective biobehavioral indicators for assessing the levels of comorbid depression. This accurate assessment of ER abilities and the level of depression, when followed by targeted intervention, could effectively improve their mental status, quality of life and social engagement, which holds practical significance.

## Author Contributions

Study concept and design: Kailing Huang, Haojun Yang, and Li Feng. Collecting data: Kailing Huang, Haojun Yang, and Qiong Zhang. Data analysis: Kailing Huang and Haojun Yang. Manuscript writing: Kailing Huang and Haojun Yang. Manuscript revising: Haojun Yang.

## Ethics Statement

This study was approved by the ethics committee of Xiangya Hospital of Central South University (No. 201912528). We confirm that we have read the Journal's position on issues involved in ethical publication and affirm that this report is consistent with those guidelines.

## Consent

All data were confidential. Participants provided informed consent before participating in the study and were allowed to withdraw from the study at any phase.

## Conflicts of Interest

The authors declare no conflicts of interest.

## Supporting information


**Table S1:** Specific values of eye movement indicators of between‐group analysis.
**Table S2:** Bivariate correlation analysis of BDI and evaluating index.
**Table S3:** Bivariate correlation analysis of the number of ASMs and evaluating indicators.

## Data Availability

The raw data supporting the conclusions of this article will be made available by the authors with a requirement.
