# Supplementary material for: More Fixations in Static Facial Regions During Emotion Recognition Among TLE Patients With Severe Depression
Source: CNS Neurosci Ther. 2025 Oct 29;31(10):e70636. doi: 10.1111/cns.70636 (PMC12569610; doi:10.1111/cns.70636)
Supplement: Supplementary file 1 — Table S1: Specific values of eye movement indicators of between‐group analysis. Table S2: Bivariate correlation analysis of BDI and evaluating index. Table S3: Bivariate correlation analysis of the number of ASMs and evaluating indicators. [file CNS-31-e70636-s001.docx]

Supplementary Table 1. Specific values of eye movement indicators of between-group analysis.

| Indicators | | Group 1 | Group 2 | Group 3 | Group 4 | P_12_/P_13_/P_14_/P_23_/P_24_/P_34_ |
| --- | --- | --- | --- | --- | --- | --- |
| **Fixation%** | |  |  |  |  |  |
| Angry | Eyes | 0.34±0.218 | 0.30±0.175 | 0.31±0.224 | 0.26±0.164 | 0.535/0.663/0.228/0.884/0.590/0.502 |
|  | Nose | 0.30±0.140 | 0.30±0.146 | 0.26±0.199 | 0.38±0.161 | 0.980/0.344/0.103/0.435/0.156/0.200 |
|  | Mouth | 0.18±0.147 | 0.23±0.174 | 0.14±0.136 | 0.17±0.128 | 0.204/0.308/0.818/0.057/0.224/0.528 |
| Disgust | Eyes | 0.32±0.194 | 0.26±0.192 | 0.30±0.187 | 0.24±0.181 | 0.269/0.782/0.189/0.492/0.810/0.373 |
|  | Nose | 0.26±0.125 | 0.30±0.107 | 0.22±0.168 | 0.37±0.135 | 0.398/0.244/**0.044**/0.093/0.098/**0.009** |
|  | Mouth | 0.24±0.168 | 0.29±0.193 | 0.21±0.164 | 0.25±0.167 | 0.294/0.456/0.850/0.134/0.495/0.446 |
| Happy | Eyes | 0.29±0.165 | 0.26±0.182 | 0.30±0.206 | 0.22±0.134 | 0.430/0.974/0.187/0.495/0.616/0.253 |
|  | Nose | 0.30±0.123 | 0.29±0.100 | 0.23±0.168 | 0.37±0.155 | 0.706/0.084/0.066/0.246/0.061/**0.022** |
|  | Mouth | 0.22±0.133 | 0.29±0.194 | 0.20±0.175 | 0.24±0.126 | 0.119/0.598/0.643/0.083/0.389/0.414 |
| Sad | Eyes | 0.33±0.195 | 0.27±0.205 | 0.34±0.235 | 0.25±0.163 | 0.306/0.830/0.184/0.303/0.747/0.194 |
|  | Nose | 0.28±0.119 | 0.29±0.116 | 0.25±0.176 | 0.38±0.165 | 0.823/0.491/0.087/0.442/0.320/0.054 |
|  | Mouth | 0.19±0.130 | 0.27±0.173 | 0.16±0.139 | 0.22±0.165 | 0.251/0.479/0.486/0.137/0.293/0.246 |
| **Fixation Count** | |  |  |  |  |  |
| Angry | Eyes | 2.11±1.517 | 1.66±1.151 | 2.08±2.005 | 1.81±1.529 | 0.305/0.946/0.526/0.427/0.779/0.629 |
|  | Nose | 1.84±0.949 | 1.58±0.839 | 1.46±1.037 | 2.52±1.363 | 0.379/0.202/**0.028**/0.724/**0.010**/**0.004** |
|  | Mouth | 1.07±0.822 | 1.21±0.861 | 0.77±0.770 | 1.19±1.193 | 0.578/0.244/0.667/0.149/0.938/0.192 |
| Disgust | Eyes | 1.94±1.351 | 1.46±1.349 | 1.75±1.240 | 1.47±1.317 | 0.202/0.624/0.235/0.517/0.994/0.541 |
|  | Nose | 1.59±0.850 | 1.52±0.760 | 1.19±0.835 | 2.22±1.185 | 0.767/0.115/**0.021**/0.274/**0.027**/**0.002** |
|  | Mouth | 1.36±0.932 | 1.54±0.964 | 1.01±0.716 | 1.47±1.106 | 0.512/0.189/0.716/0.099/0.830/0.171 |
| Happy | Eyes | 1.83±1.220 | 1.57±1.186 | 1.77±1.539 | 1.47±1.175 | 0.469/0.872/0.338/0.642/0.809/0.496 |
|  | Nose | 1.79±0.799 | 1.62±0.820 | 1.28±0.860 | 2.56±1.298 | 0.516/0.057/**0.036**/0.278/**0.025**/**0.000** |
|  | Mouth | 1.36±0.857 | 1.55±1.001 | 1.00±0.755 | 1.54±0.885 | 0.460/0.149/0.494/0.068/0.995/0.082 |
| Sad | Eyes | 2.09±1.376 | 1.70±1.430 | 2.09±1.639 | 1.64±1.482 | 0.338/0.998/0.302/0.425/0.907/0.381 |
|  | Nose | 1.76±0.789 | 1.71±0.799 | 1.36±0.797 | 2.63±1.396 | 0.844/0.130/**0.013**/0.261/**0.030**/**0.001** |
|  | Mouth | 1.20±0.863 | 1.46±0.897 | 0.86±0.656 | 1.44±1.167 | 0.314/0.179/0.382/0.300/0.953/0.069 |

Notes: Significant differences were in bold. Group 1 to 4 represents TLE patients with no/mild/moderate/severe depression in turn. And P_12_ means p values between group 1 and 2. The other P values are similar to the above description.

Supplementary Table 2. Bivariate correlation analysis of BDI and evaluating index.

| Index |  | BDI |
| --- | --- | --- |
| MoCA | r value | -0.399 |
|  | p value | < 0.001 |
| Logical Memory | r value | -0.293 |
|  | p value | 0.004 |
| FPT | r value | -0.225 |
|  | p value | 0.030 |
| Fixation Percentage_Nose | r value | 0.272 |
|  | p value | 0.008 |
| Fixation Count_Nose | r value | 0.288 |
|  | p value | 0.005 |
| Angry_Fixation Count_Nose | r value | 0.233 |
|  | p value | 0.024 |
| Disgust_Fixation Count_Nose | r value | 0.202 |
|  | p value | 0.052 |
| Happy_Fixation Count_Nose | r value | 0.280 |
|  | p value | 0.007 |
| Sad_ Fixation Count_Nose | r value | 0.319 |
|  | p value | 0.002 |

Supplementary Table 3. Bivariate correlation analysis of the number of ASMs and evaluating indicators.

| Indicators |  | Number of ASMs |
| --- | --- | --- |
| MoCA | r value | 0.008 |
|  | p value | 0.94 |
| BDI | r value | 0.17 |
|  | p value | 0.103 |
| Total FPT score | r value | -0.06 |
|  | p value | 0.571 |
| Accuracy | r value | -0.167 |
|  | p value | 0.109 |
| Fixation Percentage_Eyes | r value | -0.067 |
|  | p value | 0.523 |
| Fixation Percentage_Nose | r value | 0.066 |
|  | p value | 0.529 |
| Fixation Percentage_Mouth | r value | -0.069 |
|  | p value | 0.512 |
| Fixation Count_Eyes | r value | -0.13 |
|  | p value | 0.216 |
| Fixation Count_Nose | r value | 0.003 |
|  | p value | 0.98 |
| Fixation Count_Mouth | r value | -0.095 |
|  | p value | 0.365 |
| Run Count_Eyes | r value | -0.098 |
|  | p value | 0.349 |
| Run Count_Nose | r value | -0.066 |
|  | p value | 0.528 |
| Run Count_Mouth | r value | -0.098 |
|  | p value | 0.348 |
